# Association between penultimate ejaculatory abstinence and sperm quality: a cross-sectional study

**DOI:** 10.3389/fendo.2024.1490399

**Published:** 2024-10-17

**Authors:** Yuting Jiang, Yueying Zhu, Qingkuo Kong, Xin Lv, Qi Xi, Yang Yu

**Affiliations:** Reproductive Medicine Center and Prenatal Diagnosis Center, The First Hospital of Jilin University, Changchun, China

**Keywords:** penultimate ejaculatory abstinence, semen parameters, DNA fragmentation index, ejaculatory abstinence, male fertility

## Abstract

**Background:**

Ejaculatory abstinence (EA) duration influences semen parameters. However, the impact of penultimate ejaculatory abstinence (PEA) on conventional and functional sperm parameters remains underexplored.

**Method:**

A cross-sectional study recruited 1,503 men from a reproductive center between November 2023 and July 2024. Each participant underwent a physical examination, completed clinical questionnaires, and provided a semen sample for analysis. Generalized linear models were adjusted for potential confounders such as EA to investigate the association between PEA and various sperm parameters. Logistic regression was used to evaluate the relationship between PEA and the risk of high sperm DNA fragmentation index (DFI), oligozoospermia, asthenozoospermia, and necrozoospermia.

**Results:**

Participants were categorized into four quartiles based on PEA duration. (Q1: 1-3 days; Q2: 4-5 days; Q3: 6-9 days; Q4: > 9 days). After adjusting for potential confounders, a significant positive linear association was found between PEA and DFI, while a significant negative linear association was observed with progressive sperm motility. The longest PEA duration (Q4) correlated positively with semen concentration (*P* = 0.025), total sperm count (*P* < 0.001), and sperm vitality (*P* < 0.001). Compared to Q1, a PEA of > 9 days (Q4) was associated with higher risks of sperm DFI > 30% (adjusted odds ratio [OR] = 4.25; 95% confidence interval [CI]: 2.37-7.62), asthenozoospermia (adjusted OR = 1.45; 95% CI: 1.07-1.96), and necrozoospermia (adjusted OR = 1.99; 95% CI: 1.07-3.69). Moreover, the risk of sperm DFI > 15% was higher in Q2, Q3, and Q4 compared to Q1.

**Conclusion:**

Prolonged PEA adversely affects sperm DFI, progressive motility, and sperm vitality, increasing the likelihood of asthenozoospermia, necrozoospermia, and elevated DFI levels. These findings suggest that both EA and PEA should be considered in fertility assessments, with shorter PEA durations potentially yielding higher quality sperm, thereby enhancing male fertility evaluation and outcomes.

## Introduction

Semen analysis is a critical component in evaluating male fertility, particularly in couples facing infertility or recurrent pregnancy loss (RPL). The sample collection process significantly influences the accuracy of semen evaluation. According to World Health Organization (WHO) guidelines, semen should be collected after an abstinence period of 2 to 7 days (1). However, this recommendation, primarily based on clinical experience, may not fully consider the nuances of semen quality and its reproductive potential, particularly in the context of assisted reproductive technologies (ART).

Research examining the effects of ejaculatory abstinence (EA) on sperm parameters has yielded inconsistent results. Prolonged EA has been linked to increased semen volume, sperm concentration, and total sperm count; however, its impact on progressive motility (PR) remains inconclusive (2–5). Conversely, very short abstinence intervals (within 4 hours) have been shown to produce higher quality sperm in men with abnormal sperm parameters (6). In ART settings, shorter EA durations are associated with reduced sperm DNA fragmentation index (DFI) and improved pregnancy outcomes in intracytoplasmic sperm injection (ICSI) procedures (5–7).

While there is growing evidence supporting the benefits of shorter EA in improving fertility, it remains uncertain whether a single short-term abstinence is sufficient to optimize sperm quality. Studies on daily sperm production indicate that 2 to 3 consecutive days of ejaculation are required to deplete sperm stored in the epididymis (8, 9). During transport and storage in the epididymis, sperm are exposed to high levels of reactive nitrogen and reactive oxygen species (ROS), impairing sperm quality (10). Incomplete emptying of the epididymis after a single ejaculation (11), means that residual sperm from previous ejaculations can influence the quality of subsequent samples (12). Therefore, the current EA and the preceding abstinence period (penultimate EA or PEA) play a role in determining semen quality. Although the precise impact of PEA is challenging to quantify and is frequently overlooked, consecutive short intervals of ejaculation could be a feasible and practical approach for couples dealing with fertility challenges or undergoing ART.

To further explore this, PEA, the interval between the two most recent ejaculations, was recorded. This study aimed to evaluate the association between PEA and sperm quality.

## Methods

### Ethical approval

This cross-sectional study was approved by the medical ethics committee of the First Hospital of Jilin University (approval number: 23K227-001) and registered on ClinicalTrials.gov (identifier: NCT06172504). The registration date was November 11, 2023, with the first subject enrolled on the same date. All participants provided written informed consent, and the study adhered to the principles of the Declaration of Helsinki.

### Patients

This study included 1,697 men seeking fertility assistance recruited between November 2023 and July 2024. Participants self-reported fertility concerns, including pre-pregnancy examinations or infertility issues. All participants underwent a standard semen analysis as part of their assessment. Eligible participants were aged between 22 and 45 years, and adhered to an EA period of 2 to 7 days. Exclusion criteria included severe oligospermia (sperm concentration < 1×10^6^/mL), azoospermia, urogenital infections (such as epididymitis and orchitis), previous genital surgery (such as testicular cancer, cryptorchidism, and testicular torsion), moderate to severe varicocele, and occupational exposure to high temperatures, chemicals, or radiation. Men with a PEA exceeding 4 weeks were also excluded. Ultimately, 1,503 men were enrolled in this study (**Supplementary Figure 1**).

### Questionnaires

Participants completed questionnaires covering demographic data (age, height, weight, educational level, occupation, and residence), lifestyle factors (alcohol consumption, smoking), medical history (urogenital infections and genital surgeries), and details about their female partner’s pregnancy and delivery history. Body mass index (BMI) was calculated by dividing weight (in kilograms) by height squared (in meters). The questionnaire also included inquiries about the participants’ awareness of the effects of EA on sperm quality, whether the couple used intercourse timing strategies during ovulation, the number of ejaculations during the ovulatory period, and the frequency of ejaculations outside of this window. Questionnaires were completed before the disclosure of semen analysis results.

### Semen collection and analysis

The semen collection and analysis procedures have been detailed in our previous study (13). Semen samples were obtained through masturbation into pre-weighed, sterile containers following 2 to 7 days of abstinence. The container was reweighed after collection, and semen volume was calculated by subtracting the initial weight using the formula: semen volume (mL) = weight (g)/concentration (g/mL). Before conducting conventional semen analysis, samples were incubated at 37°C until liquefaction. Sperm concentration, motility, and vitality were assessed in 5 mL of semen using a computer-assisted semen analysis (CASA) system (BEION S3, Beion Medical Technology Co. Ltd., Shanghai, China). PR and non-progressive motility (NP) were measured, and total sperm count was calculated as sperm concentration (× 10^6^/mL) × volume (mL). The PR sperm count was determined as the total sperm count (× 10^6^/ejaculate) × PR%. Semen parameters were classified according to WHO guidelines (14). Sperm morphology was evaluated during the initial semen analysis, as described in our previous study (15). An aliquot of 5 to 10 μL of semen, depending on sperm concentration, was placed on a pre-cleaned slide and stained using the Diff-Quik staining protocol (Anke Biotechnology [Group] Co. Ltd., Anhui, China). Two qualified technicians, nationally certified in semen analysis and experienced in teratozoospermia assessment, independently evaluated 200 sperm cells.

The sperm DNA fragmentation index (DFI) was measured using a sperm DNA staining solution (Anke Biotechnology [Group] Co. Ltd., Anhui, China) (16). employing a fluorescence staining technique with flow cytometry (Mindray Bio-Medical Electronics Co., Ltd., Shenzhen, China). A minimum of 5,000 spermatozoa per sample were counted and analyzed. Results were reported as the percentage of sperm with fragmented DNA (% DNA fragmentation), calculated through flow cytometer software.

### Statistical analyses

Statistical analyses were conducted using R software (The R Foundation, version 4.3.0) and EmpowerStats software (www.empowerstats.net; X&Y Solutions, Inc., Boston, Massachusetts, version 4.2) (17, 18). Baseline characteristics were presented as mean ± standard deviation (SD) for continuous variables or as counts and percentages (n, %) for categorical variables. Differences across quartiles of PEA were evaluated using the Kruskal–Wallis test for continuous variables and the Chi-square or Fisher’s exact test for categorical data. Spearman correlation was applied to examine the relationship between PEA and clinical characteristics (including sperm parameters). Each semen parameter was analyzed by quartile of PEA, with the lowest quartile (Q1) as the reference group. Multiple linear regression models were employed to adjust for potential confounders, including age, BMI, smoking status, alcohol consumption, education level, abstinence duration, cohabitation duration, and the number of pregnancy losses for factors possibly affecting semen parameters. Logistic regression models assessed the risk relationships between PEA and high DFI levels (> 15%, > 30%), oligozoospermia, asthenozoospermia, and necrozoospermia. WHO guidelines define the lower reference limits for semen parameters: a total sperm count < 39×10^6^/mL indicates oligozoospermia, PR sperm < 32% signifies asthenozoospermia, and sperm vitality < 58% denotes necrozoospermia. While no standardized thresholds exist for sperm DFI, levels greater than 15% or 30% are commonly used to define high DFI (7, 19). Statistical significance was set at *P*-value < 0.05.

## Results

A total of 81.4% of couples were unaware of EA’s impact on sperm quality. Furthermore, 44.6% of couples timed intercourse during the ovulatory period, with only 12.6% having intercourse 1-2 times during ovulation and rarely engaging outside this period.

### Correlation between PEA and semen parameters

**Table 1** presents the correlation analysis between PEA and various clinical factors. A longer PEA was significantly associated with older age (*r* = 0.08, *P* = 0.003) and a higher number of pregnancy losses (*r* = 0.06, *P* = 0.028). PEA was positively correlated with sperm DFI (*r* = 0.22, *P* < 0.001), semen volume (*r* = 0.07, *P* = 0.011), sperm concentration (*r* = 0.07, *P* = 0.010), and total sperm count (*r* = 0.11, *P* < 0.001). In contrast, it was negatively correlated with sperm PR (*r* = -0.06, *P* = 0.033) and vitality (*r* = -0.11, *P* < 0.001).

**Table 1:** Correlation between penultimate ejaculatory abstinence and semen parameters.

Clinical characteristics	PEA	
	*r* (95% CI)	*P* value
Age	0.08 (0.03-0.13)	0.003
BMI	0.02 (-0.03-0.07)	0.504
Smoke	0.00 (-0.05-0.05)	0.870
Alcohol	0.02 (-0.04-0.07)	0.560
Duration of cohabitation	0.03 (-0.02-0.08)	0.304
Number of pregnancies	0.05 (-0.00-0.10)	0.074
Number of pregnancy losses	0.06 (0.01-0.11)	0.028
DFI	0.22 (0.17-0.27)	< 0.001
Semen volume	0.07 (0.02-0.12)	0.011
Sperm concentration	0.07 (0.02-0.12)	0.010
Progressive motility	-0.06 (-0.11-0.00)	0.033
Sperm vitality	-0.11 (-0.16–0.06)	< 0.001
Total sperm count	0.11 (0.06-0.16)	< 0.001
Sperm morphology	-0.04 (-0.09-0.01)	0.144

PEA, penultimate ejaculatory abstinence (days); CI, confidence interval; BMI, body mass index; DFI, DNA fragmentation index.

### Comparison of clinical characteristics across PEA quartiles

**Table 2** presents the semen samples categorized into four quartiles based on PEA. An increase in PEA was associated with advancing age (*P* = 0.004). Although significant differences in abstinence duration were observed across the quartiles (*P* = 0.001), the pattern did not follow a consistent trend. The incidence of pregnancy losses was higher in the Q3 and Q4 groups compared to Q1 and Q2. Moreover, sperm DFI (*P* < 0.001), semen volume (*P* = 0.007), sperm concentration (*P* = 0.050), and total sperm count (*P* = 0.001) increased with longer PEA, while sperm PR (*P* = 0.010) and vitality (*P* < 0.001) decreased as PEA increased.

**Table 2:** Comparison of clinical characteristics across different penultimate ejaculatory abstinence quartiles.

Clinical characteristics	All men N=1503	PEA quartile (days)				*P* value
		Q1 (1-3) N=382	Q2 (4-5) N=396	Q3 (6-9) N=376	Q4 (>9) N=349	
Age (years)	32.3 ± 4.1	31.9 ± 4.1	32.2 ± 4.0	32.3 ± 4.1	33.0 ± 4.0	0.004
BMI (kg/m^2^)	26.2 ± 4.1	26.0 ± 4.1	26.4 ± 4.1	25.9 ± 4.0	26.3± 4.1	0.393
Smokers, n (%)	594 (39.5)	156 (40.8)	160 (40.4)	135 (35.9)	143 (41.0)	0.428
Alcohol consumer (>2 times/week), n (%)	111 (7.4)	32 (8.4)	22 (5.6)	27 (7.2)	30 (8.6)	0.353
Education level, n (%)						0.457
No higher than university	358 (23.8)	93 (24.3)	92 (23.2)	80 (21.3)	93 (26.6)	
University	991 (65.9)	242 (63.4)	266 (67.2)	260 (69.1)	223 (63.9)	
University above	154 (10.2)	47 (12.3)	38 (9.6)	36 (9.6)	33 (9.5)	
Duration of cohabitation (years)	4.2 ± 3.3	4.1 ± 3.2	4.1 ± 3.2	4.1± 3.2	4.5 ± 3.6	0.354
Abstinence time (days)	4.2 ± 1.2	4.1 ± 1.2	4.3 ± 1.3	4.3 ± 1.3	4.0 ± 1.1	0.001
No. of pregnancy losses (n)	0.5 ± 0.8	0.4 ± 0.7	0.4 ± 0.8	0.5 ± 0.8	0.5 ± 0.8	0.017
DFI, (%)	15.1 ± 9.3	12.78 ± 7.2	14.5 ± 7.7	15.1 ± 8.9	18.4 ± 12.2	< 0.001
Semen volume (mL)	3.7 ± 1.5	3.5 ± 1.3	3.6 ± 1.3	3.9 ± 1.5	3.8 ± 1.6	0.007
Sperm concentration (millions/mL)	65.6 ± 39.7	63.7 ± 38.0	64.1 ± 39.6	64.3 ± 39.3	70.9 ± 41.8	0.050
Sperm PR (%)	32.7 ± 15.6	34.9 ± 15.8	32.0 ± 15.6	32.3 ± 16.0	31.3 ± 15.0	0.010
Sperm vitality (%)	74.2 ± 10.4	76.0 ± 9.7	74.5 ± 9.1	74.0 ± 10.8	72.1 ± 11.8	< 0.001
Total sperm count (millions)	231.6 ± 148.0	215.4 ± 134.7	217.3 ± 134.7	236.5 ± 150.2	260.2 ± 168.2	0.001
Total PR sperm count (millions)	77.3 ± 62.2	78.2 ± 65.6	71.9 ± 58.5	79.8 ± 64.8	79.7± 59.5	0.229
Sperm morphology (%)	2.2 ± 1.2	2.2 ± 1.2	2.2 ± 1.3	2.1 ± 1.3	2.1 ± 1.2	0.424

Mean ± standard deviation (SD) for continuous variables; P-values were calculated using weighted linear regression models.

n (%) for categorical variables; P-values calculated using weighted chi-square tests.

PEA, penultimate ejaculatory abstinence (days); BMI, body mass index; DFI, DNA fragmentation index; PR, progressive motility; SD, standard deviation; Q1-Q4 represent PEA quartiles.

### Adjusted regression coefficients of PEA for semen parameters

After adjusting for confounding factors (**Table 3**), significant associations were identified between PEA and sperm DFI across quartiles (Q2, *P* = 0.022; Q3, *P* = 0.002; and Q4, *P* < 0.001). Conversely, a substantial negative linear association was found between PEA and sperm PR (Q2, *P* = 0.016; Q3, *P* = 0.025; Q4, *P* = 0.002). The longest PEA quartile (Q4) showed a positive correlation with semen concentration (*P* = 0.025) and total sperm count (*P* < 0.001). Moreover, PEAs in Q3 and Q4 were positively associated with semen volume (Q3, *P* = 0.001; Q4, *P* < 0.001) and negatively associated with sperm vitality (Q3, *P* = 0.008; Q4, *P* < 0.001).

**Table 3:** Adjusted regression coefficients for the association between penultimate ejaculatory abstinence and semen parameters.

Semen parameter	Q1(1-3 days)	Q2 (4-5 days)		Q3 (6-9 days)		Q4 (>9 days)	
		*β* (95% CI)	*P* value	*β* (95% CI)	*P* value	*β* (95% CI)	*P* value
DFI							
Crude	Reference	1.74 (0.46-3.02)	0.008	2.32 (1.03-3.62)	<0.001	5.63(4.31-6.95)	< 0.001
Adjusted	Reference	1.47 (0.22-2.73)	0.022	2.00 (0.72-3.28)	0.002	5.38(4.07-6.69)	< 0.001
Semen volume							
Crude	Reference	0.10 (-0.10-0.30)	0.321	0.37 (0.16-0.57)	<0.001	0.32(0.11-0.53)	0.003
Adjusted	Reference	0.07 (-0.13-0.27)	0.525	0.33 (0.13-0.54)	0.001	0.39(0.18-0.60)	< 0.001
Sperm concentration							
Crude	Reference	0.41 (-5.16-5.98)	0.885	0.63 (-5.02-6.27)	0.828	7.21 (1.46-12.97)	0.014
Adjusted	Reference	-1.12 (-6.62-4.39)	0.690	-0.96 (-6.56-4.63)	0.736	6.56 (0.83-12.29)	0.025
Sperm PR							
Crude	Reference	-2.90 (-5.09–0.71)	0.010	-2.59 (-4.81–0.37)	0.022	-3.63 (-5.89–1.37)	0.002
Adjusted	Reference	-2.71 (-4.90–0.51)	0.016	-2.56 (-4.79–0.32)	0.025	-3.67 (-5.96–0.01)	0.002
Sperm vitality							
Crude	Reference	-1.44 (-2.89-0.01)	0.052	-2.03 (-3.50–0.01)	0.007	-3.84 (-5.34–0.02)	< 0.001
Adjusted	Reference	-0.01 (-2.79-0.12)	0.073	-1.99 (-3.47–0.51)	0.008	-3.91 (-5.42–0.02)	< 0.001
Total sperm count							
Crude	Reference	1.92 (-18.74-22.59)	0.855	21.11 (0.18-42.04)	0.048	44.87 (23.54-66.20)	< 0.001
Adjusted	Reference	-6.07 (-25.96-13.81)	0.550	13.19 (-7.01-33.40)	0.201	47.15 (26.45-67.85)	< 0.001
Total PR sperm count							
Crude	Reference	-6.30 (-15.05-2.44)	0.158	1.58 (-7.27-10.44)	0.727	1.46 (-7.57-10.48)	0.752
Adjusted	Reference	-8.23 (-16.85-0.40)	0.062	-0.56 (-9.33-8.20)	0.900	2.10 (-6.88-11.07)	0.647
Sperm morphology							
Crude	Reference	-0.08 (-0.25-0.09)	0.372	-0.09 (-0.27-0.08)	0.297	-0.12 (-0.30-0.06)	0.205
Adjusted	Reference	-0.08 (-0.25-0.09)	0.370	-0.10 (-0.28-0.08)	0.275	-0.13 (-0.31-0.05)	0.161

Crude: unadjusted model; adjusted: P-values derived from multiple linear regression models adjusting for age, BMI, smoking, alcohol consumption, education level, abstinence duration, cohabitation duration, and number of pregnancy losses.

PEA, penultimate ejaculatory abstinence; BMI, body mass index; DFI, DNA fragmentation index; PR, progressive motility; CI, confidence interval. Q1-Q4 represent PEA quartiles.

### Odds ratio (95% confidence interval) of PEA’s impact on abnormal semen parameters

**Figure 1:**
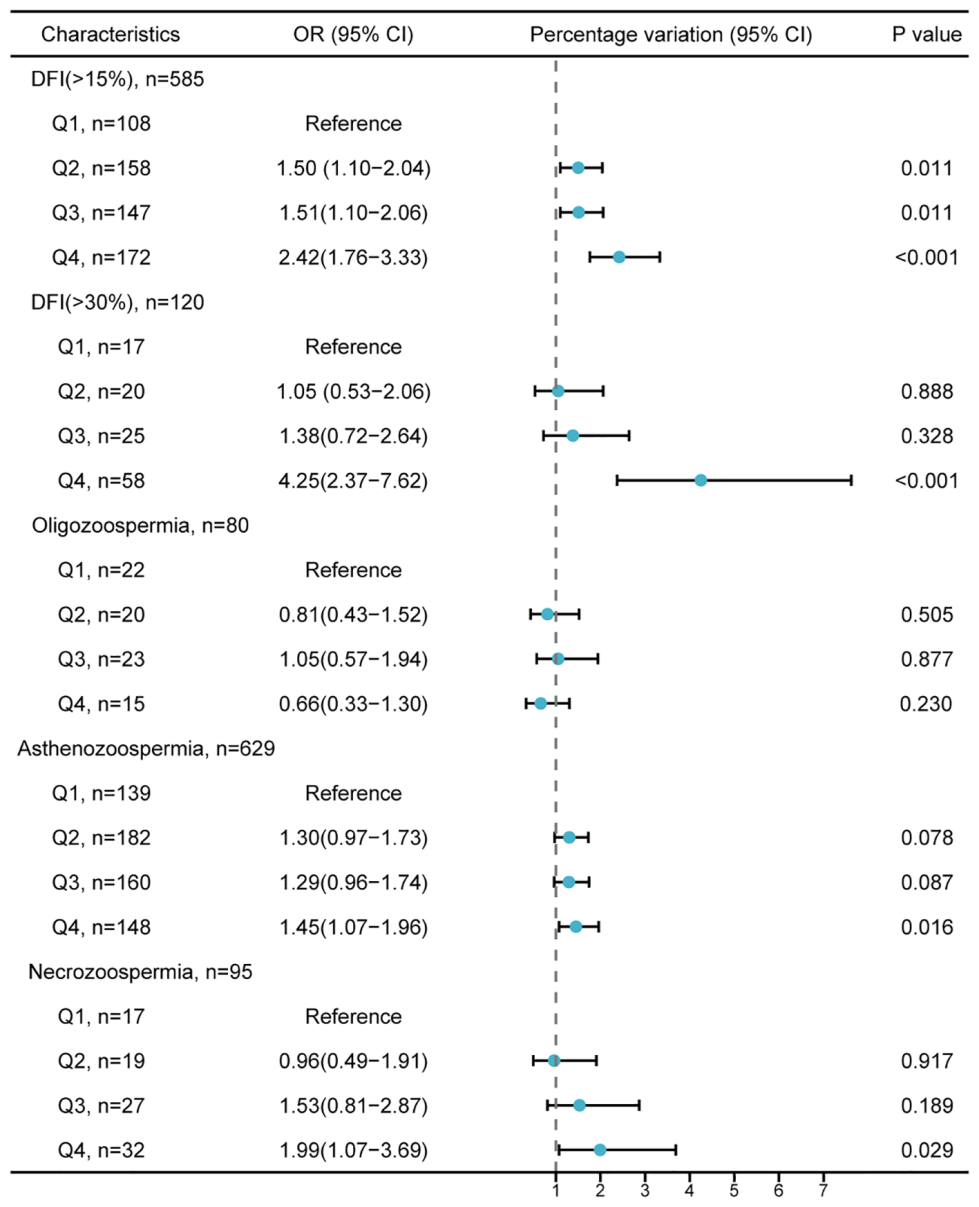
Adjusted odds ratio (95% confidence intervals) for the impact of penultimate ejaculatory abstinence on abnormal semen parameter values. The lower reference values for semen parameters follow the World Health Organization guidelines: total sperm count < 39×10^6^/mL (oligozoospermia), progressive motility < 32% (asthenozoospermia), and vitality < 58% (necrozoospermia). The model is adjusted for age, BMI, smoking, alcohol consumption, education level, abstinence duration, cohabitation duration, and number of pregnancy losses. PEA, penultimate ejaculatory abstinence; DFI, DNA fragmentation index; OR, odds ratio; CI, confidence interval; BMI, body mass index.

The odds of having a sperm DFI > 15% were significantly higher in Q2 (adjusted OR = 1.5, 95% CI: 1.10-2.04), Q3 (adjusted OR = 1.51; 95% CI: 1.10-2.06), and Q4 (adjusted OR = 2.42; 95% CI: 1.76-3.33) compared to Q1 (**Figure 1**). In Q4, there was also an increased risk of sperm DFI > 30% (adjusted OR = 4.25; 95% CI: 2.37-7.62), asthenozoospermia (adjusted OR = 1.45; 95% CI: 1.07-1.96), and necrozoospermia (adjusted OR = 1.99; 95% CI: 1.07-3.69) compared to Q1.

## Discussion

To the best of our knowledge, this is the first observational study to investigate the association between PEA and semen parameters. The effects of PEA on sperm quality appear distinct from those related to EA. While shorter periods of abstinence tend to result in better sperm quality, a single ejaculation may not empty the sperm from the epididymis (6). The remaining sperm, crucial for natural conception and *in-vitro* fertilization (IVF), are influenced by both EA and PEA. This study revealed that 81.4% of couples were unaware of the relationship between EA and semen quality. Moreover, 44.6% reported having intercourse during ovulation, with 12.6% engaging in intercourse 1-2 times solely during this period and rarely at other times. This observation indicates that in China, most couples are unaware of the impact of EA on sperm quality, and the potential effects of PEA are frequently overlooked. For couples experiencing RPL, emotional and psychological stress may prompt them to engage in sexual intercourse primarily during the ovulation window (20). In such cases, men may refrain from ejaculation for one to several weeks before ovulation. Although repeated sexual intercourse during ovulation might ensure sufficient EA, it could lead to prolonged PEA, which in turn may elevate fertility risks.

Previous studies on EA have primarily focused on the effects of a single ejaculation on semen parameters, whereas this study examined the impact of multiple ejaculations. Semen parameters have been examined across consecutive ejaculations within a single day or over successive days. However, they primarily focused on short-term EA, distinguishing them from the current investigation. This study highlights the previously overlooked impact of PEA on subsequent sperm quality rather than immediate effects. In this study, PEA correlated positively with age and the number of pregnancy losses. After adjusting for potential confounders, longer PEA was significantly associated with increased sperm DFI, semen volume, sperm concentration, and total sperm count. However, prolonged PEA negatively impacted sperm PR and vitality. Moreover, individuals in Q4 demonstrated a greater risk of elevated DFI, asthenozoospermia, and necrozoospermia than those in Q1.

High Sperm DFI negatively impacts male fertility, resulting in lower pregnancy and live birth rates following ART (21, 22). As sperm travel through the epididymis for storage and transport, DNA damage and fragmentation accumulate (23). Therefore, sperm from the caput and corpus regions of the epididymis already exhibit DNA damage caused by ROS before reaching the cauda epididymis during ejaculation. This damage initiates during the PEA phase and persists into the EA phase within the cauda epididymis (12). Our study revealed a linear increase in sperm DFI with extended PEA after adjusting for confounding factors. This observation suggests that DNA damage may occur early in the process or become more susceptible to rapid deterioration during transportation or storage, potentially due to decreased DNA repair capacity during the later stages of spermatogenesis. Furthermore, PEA increased with paternal age, resulting in more significant sperm DNA damage and fragmentation (24). This finding indicates that advanced paternal age exacerbates sperm DFI, both due to aging and prolonged periods without ejaculation. Understanding the ejaculatory patterns of older men is critical to minimizing these impacts on sperm DFI. Moreover, prolonged PEA was associated with a higher risk of elevated sperm DFI. When PEA exceeded 3 days, the likelihood of sperm DFI > 15% increased. For PEA longer than 9 days, the risk more than doubled compared to 3 days or less, and the risk of DFI > 30% more than quadrupled. In clinical practice, the European Society of Human Reproduction and Embryology (ESHRE) recommends assessing sperm DFI in couples experiencing RPL for diagnostic purposes (25). Therefore, for couples facing RPL, greater attention should be paid to male ejaculatory habits. Men should have multiple ejaculations before the ovulation period. Based on our data, ejaculating every three may result in higher quality sperm. For men who have abstained for an extended period, it is recommended to aim for at least two ejaculations within a few days before ovulation to minimize the risk of pregnancy loss.

In this study, conventional semen parameters were examined, revealing a significant increase in sperm concentration and total sperm count in the Q4 group compared to the Q1 group. Interestingly, the risk of oligospermia did not rise with prolonged PEA, contrary to earlier studies. For instance, Comar et al. reported significantly higher sperm concentrations in groups with longer ejaculatory abstinence (2-5 days and > 5 days) compared to those with < 2 days (26). This discrepancy suggests that PEA may have less influence on sperm quantity than EA. However, a significant negative linear correlation was observed between PEA and sperm PR, possibly due to variations in epididymal transit duration. The cauda epididymis is responsible for sperm defense, immune responses, and fertilization (27); it is considerably influenced by seminal fluid stasis, the accumulation of aging sperm, and the clearance of these sperm cells (28). This process can negatively impact both PR and sperm vitality. When PEA exceeded 5 days (Q3 and Q4), there was a significant increase in dead sperm. After adjusting for confounding factors, the risks of asthenozoospermia and necrozoospermia were significantly higher in the Q4 group than in the Q1 group.

**Figure 2:**
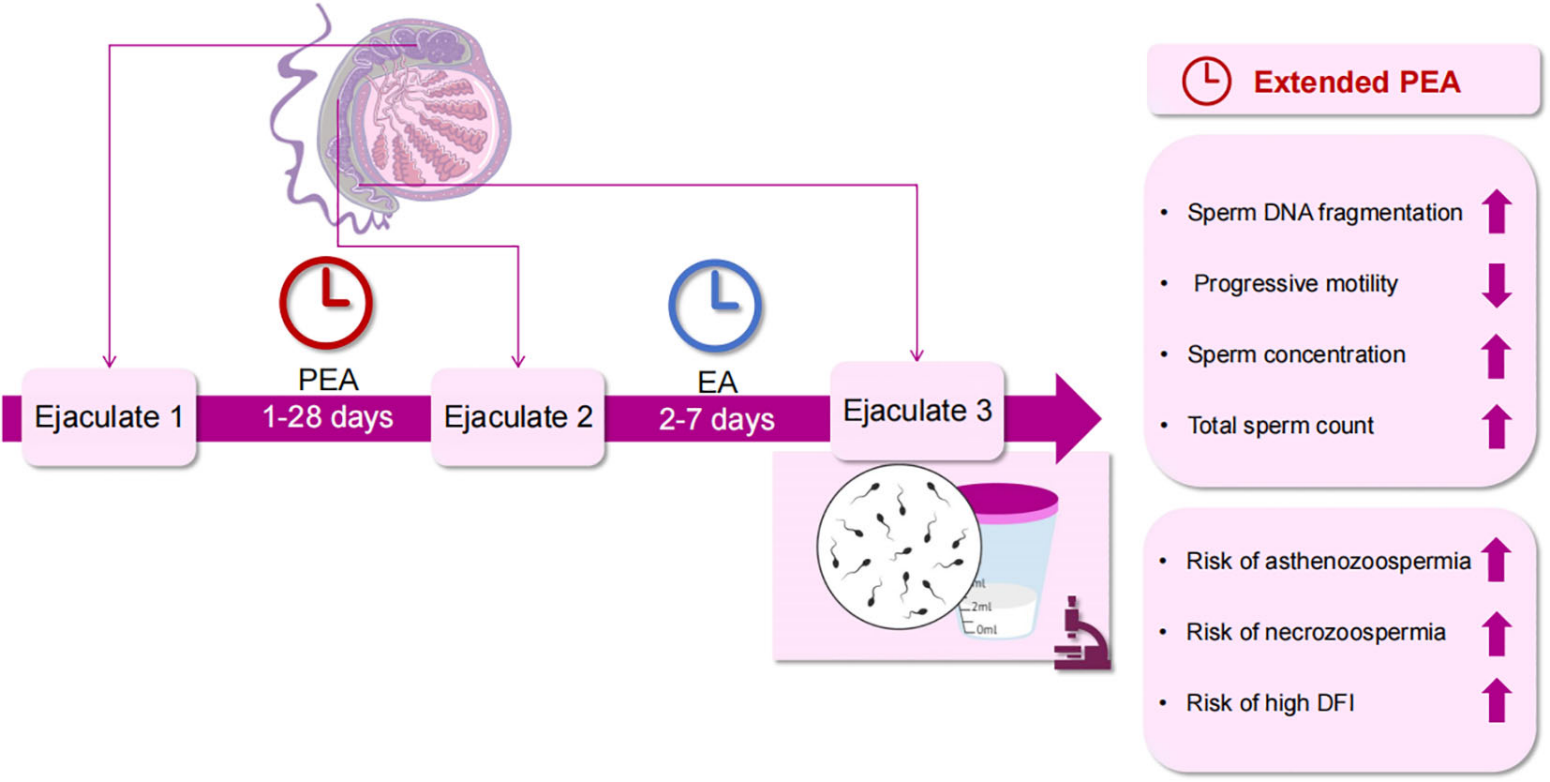
Effects of PEA on sperm quality. PEA, penultimate ejaculatory abstinence; EA, ejaculatory abstinence; DFI, DNA fragmentation index.

These findings indicate that PEA > 5 days adversely affects semen quality by increasing sperm DFI and reducing PR and vitality despite an increase in sperm concentration and total sperm count. The total PR sperm count remained unchanged. This study’s main findings are depicted in **Figure 2**. Moreover, PEA exceeding 9 days elevates the risk of high DFI, asthenozoospermia, and necrozoospermia. Therefore, it is crucial to inquire about the timing of recent ejaculations during medical history collection. Consequently, it is recommended to inquire about the timing of the last two ejaculations during medical history collection, allowing for a more accurate evaluation of semen parameters and male fertility by considering EA and PEA. For individuals with infrequent ejaculation, reducing PEA presents a practical and personalized approach to improving sperm quality.

This study’s strengths include its substantial sample size, which facilitates a robust evaluation of the relationship between PEA and semen parameters. Moreover, it accounts for potential confounding factors such as EA, age, BMI, alcohol and smoking habits, cohabitation duration, and number of pregnancies and pregnancy losses.

However, the study has some limitations. Firstly, using samples from a single center may restrict the ability to detect correlations in adjusted analyses. Secondly, there is a risk of recall bias associated with reporting prolonged PEA. Future research should emphasize prospective randomized controlled study designs to yield more accurate and reliable data.

In conclusion, this study demonstrates that prolonged PEA adversely affects sperm DFI, PR, and sperm vitality. Moreover, it increases the risks of high DFI, asthenozoospermia, and necrozoospermia. These frequently overlooked findings can significantly influence reproductive outcomes. Therefore, it is crucial to consider both the current EA and the PEA when evaluating semen parameters and their implications for fertility. Shorter PEA intervals are likely to produce higher quality sperm, which could enhance male fertility assessment and improvement efforts.

## Data Availability

The original contributions presented in the study are included in the article/**Supplementary Material**, Further inquiries can be directed to the corresponding author.

## Supplementary material

Supplementary Figure 1 Flow diagram illustrating the selection process of the study population.
