# Supplementary figures and images for: Association between penultimate ejaculatory abstinence and sperm quality: a cross-sectional study
Source: Front Endocrinol (Lausanne). 2024 Oct 17;15:1490399. doi: 10.3389/fendo.2024.1490399 (PMC11524872; doi:10.3389/fendo.2024.1490399)

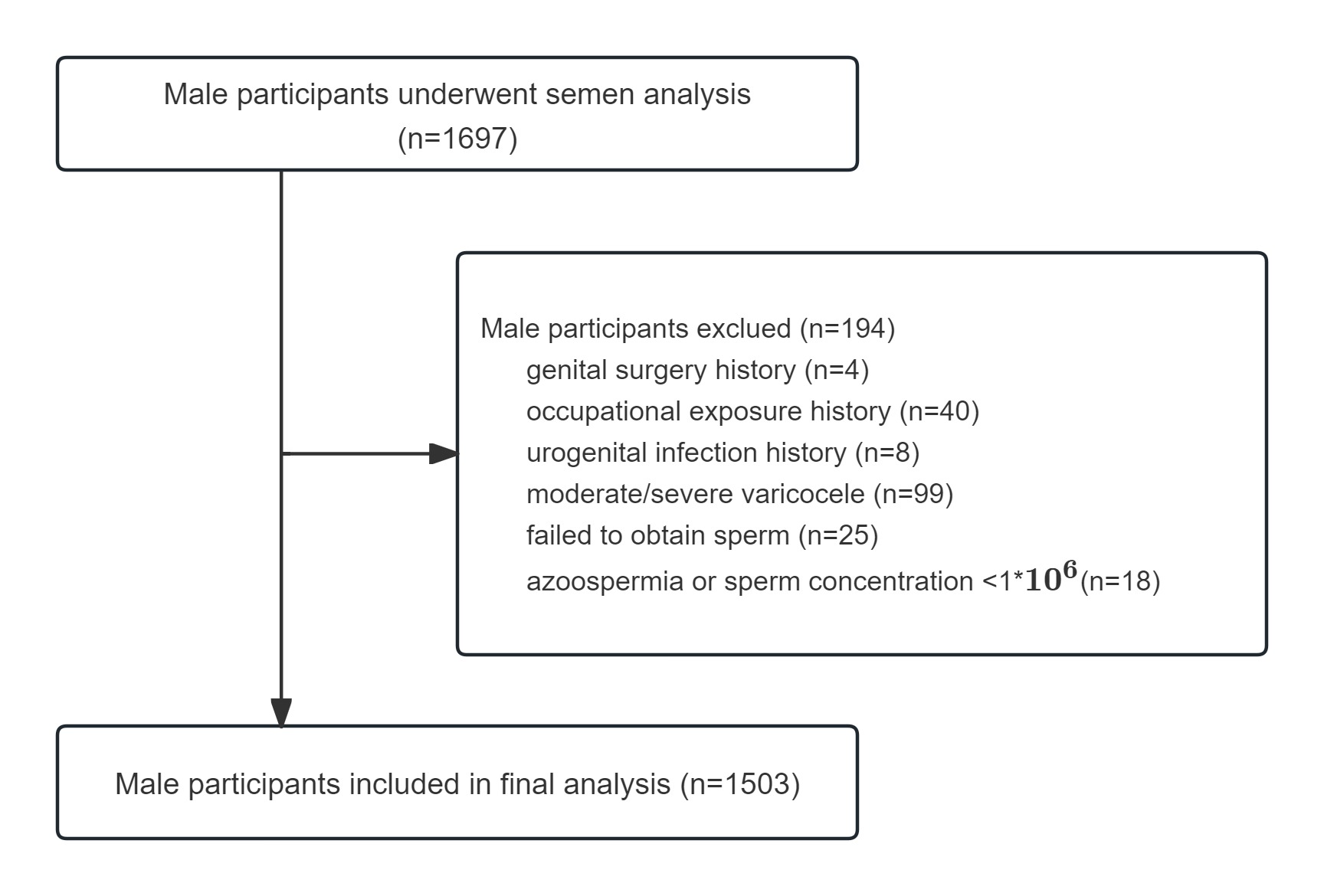

Supplement: Supplementary Figure 1 — Flow diagram illustrating the selection process of the study population. [file Image1.jpeg]
